# Dietary Acrylamide Intake and the Risk of Hematological Malignancies: The Japan Public Health Center-Based Prospective Study

**DOI:** 10.3390/nu13020590

**Published:** 2021-02-11

**Authors:** Ling Zha, Rong Liu, Tomotaka Sobue, Tetsuhisa Kitamura, Junko Ishihara, Ayaka Kotemori, Sayaka Ikeda, Norie Sawada, Motoki Iwasaki, Shoichiro Tsugane, for the JPHC Study Group

**Affiliations:** 1 Division of Environmental Medicine and Population Sciences, Department of Social and Environmental Medicine, Graduate School of Medicine, Osaka University, 2-2 Yamadaoka, Suita 565-0871, Japan; ivy_mist@outlook.com (L.Z.); lucky_unatan@yahoo.co.jp (T.K.); sayakaikeda0201@gmail.com (S.I.); 2 Department of Epidemiology and Biostatistics, School of Public Health, Xuzhou Medical University, Xuzhou 221004, China; liur8939@163.com; 3 Department of Food and Life Science, School of Life and Environmental Science, Azabu University, 1-17-71 Fuchinobe, Chuo-ku, Sagamihara, Kanagawa 252-5201, Japan; j-ishihara@azabu-u.ac.jp (J.I.); kotemori@azabu-u.ac.jp (A.K.); 4 Epidemiology and Prevention Group, Center for Public Health Sciences, National Cancer Center, 5-1-1 Tsukiji, Chuo-ku, Tokyo 104-0045, Japan; nsawada@ncc.go.jp (N.S.); moiwasak@ncc.go.jp (M.I.); stsugane@ncc.go.jp (S.T.)

**Keywords:** acrylamide, malignant lymphoma, multiple myeloma, leukemia, cohort

## Abstract

Acrylamide, which is present in many daily foods, is a probable human carcinogen. In 2002, it was identified in several common foods. Subsequently, western epidemiologists began to explore the relationship between dietary acrylamide exposure and cancer risk; however, limited suggestive associations were found. This prospective study aimed to examine the association between dietary acrylamide intake and the risk of hematological malignancies, including malignant lymphoma (ML), multiple myeloma (MM), and leukemia. We enrolled 85,303 participants in the Japan Public Health Center-based Prospective study on diet and cancer as from 1995. A food frequency questionnaire that included data on acrylamide in all Japanese foods was used to assess dietary acrylamide intake. We applied multivariable adjusted Cox proportional hazards models to reckon hazard ratios (HRs) for acrylamide intake for both categorical variables (tertiles) and continuous variables. After 16.0 median years of follow-up, 326 confirmed cases of ML, 126 cases of MM, and 224 cases of leukemia were available for final multivariable-adjusted analysis. HRs were 0.87 (95% confidence interval [CI]: 0.64–1.18) for ML, 0.64 (95% CI: 0.38–1.05) for MM, and 1.01 (95% CI: 0.71–1.45) for leukemia. Our results implied that acrylamide may not be related to the risk of hematological malignancies.

## 1. Introduction

Acrylamide is as a chemical substance that is mainly used in industrial applications as a raw material for paper strength enhancers, soil conditioners, adhesives, pigments and paints, and cosmetics. In 1994, acrylamide was categorized as 2A (probably carcinogenic to humans) in the carcinogenicity classification by the International Agency for Research on Cancer (IARC) because there was insufficient evidence concerning its carcinogenicity to humans, but there was sufficient evidence of carcinogenicity in animal studies [1]. According to studies on rodents, there is a possibility of a positive dose-response relationship between acrylamide exposure and cancers of the breast, skin, lung, oral tissues, and thyroid gland [2,3,4]. Before the early 2000s, occupational settings were considered the main means of human exposure to acrylamide. However, epidemiological studies of the association between occupational acrylamide exposure and the risk of cancer incidence did not show positive results [5].

In 2002, Swedish researchers suggested that meals are another important source of acrylamide [6]. They found that in starchy foods, acrylamide is formed by the Maillard reaction between the amino acid asparagine and reducing sugars during high-temperature cooking [6]. Later, Western epidemiologists investigated the association between the incidence of cancers and dietary acrylamide intake. They found that only kidney cancer, postmenopausal estrogen receptor-positive breast cancer, and endometrial and ovarian cancer were associated with dietary acrylamide intake [7,8].

Recently, Japan has assessed the risks of dietary acrylamide intake for esophageal, gastric, colorectal, breast, endometrial, ovarian, lung, liver, and pancreatic cancers [9,10,11,12,13,14]. Positive results were not found, most of which were consistent with previous studies.

At present, there are only two western studies that have published their findings on the association between hematological malignancies and dietary acrylamide intake [15,16]. International comparisons have revealed geographical and ethnic variations in the incidence of hematological malignancies, and the disparities might be explained by the genetic and cumulative exposure to the environment [17,18,19,20]. The baseline risk is different in Japan and western countries. Moreover, the major sources of acrylamide estimated in the diet are different [8,14,21]. In consequence, the impact of acrylamide from different dietary sources on cancer may vary in different countries, current epidemiological evidence for hematological malignancies is inadequate. Therefore, our research aimed to determine whether dietary acrylamide intake is associated with the development of hematological malignancies including malignant lymphoma (ML), multiple myeloma (MM), and leukemia, making use of a large-scale population-based cohort study in Japan.

## 2. Materials and Methods

### 2.1. Study Cohort and Participants

This study was based on the Japan Public Health Center-based Prospective Study (JPHC Study, including Cohorts I and II), and its detailed design has been described elsewhere [22]. Eleven public health centers with 140,420 inhabitants, aged 40–69 years old, across Japan were included in the JPHC study. A self-administered lifestyle questionnaire was applied to acquire information from the inhabitants in the baseline survey conducted during 1990 to 1993. Five years later, a follow-up survey was conducted. More detailed information regarding the frequency of food and beverage intake was extracted. The validity and reproducibility of the self-administered food frequency questionnaire (FFQ) from the five-year follow-up survey were evaluated and found to be at an appropriate level, indicating that the FFQ estimated the dietary intake of individuals accurately for epidemiological utilization. Therefore, we used the Five-year follow-up survey, during 1995 to 1998, as the starting point of the study.

**Figure 1:**
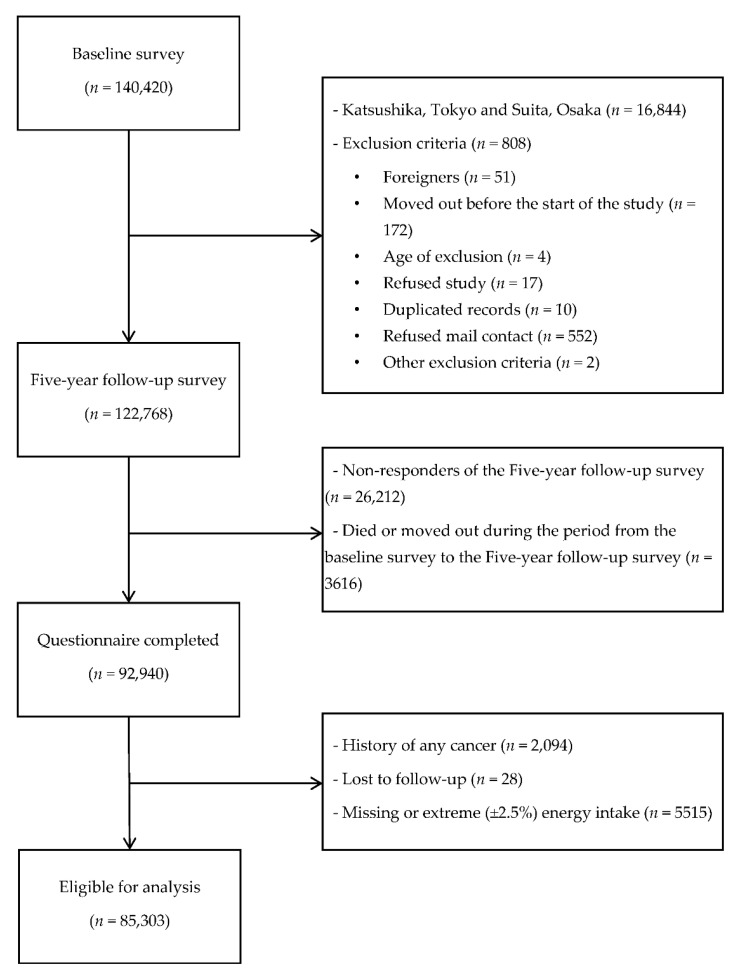
Flow diagram of the study selection.

In the present study, we excluded participants in Katsushika, Tokyo, whose data on cancer incidence were not available and those in Suita, Osaka, whose definition of the study population was different (*n* = 16,844, as shown in Figure 1). Furthermore, participants who satisfied one of the following conditions were excluded: exclusion criteria (*n* = 808), incomplete the Five-year follow-up questionnaire (*n* = 26,212), death or movement during the first five years from the baseline survey to the Five-year follow-up survey (*n* = 3616), having a cancer history (*n* = 2094), lost to follow-up (*n* = 28), and extremely low/high energy values or incomplete information on total energy (*n* = 5515).

This research was conducted following the Declaration of Helsinki Ethical Principles and Good Clinical Practices. The protocol has been approved by the Research Ethics Committee of Osaka University (approval number: 14020-9), National Cancer Center Japan (approval number: 2001–021), and Azabu University (approval number: 2527). Participants were informed of the objectives of the study, and that completion of the survey questionnaire was considered as providing consent to participate. More detailed information of the JPHC Study could be obtained from a previously publication [22].

### 2.2. Assessment of Acrylamide Intake

The FFQ was used to assess the intake of food, beverage, and nutrients. Daily consumption of 138 food and beverage items was recorded [23]. Portion sizes were divided into three categories: (1) less than 0.5 times the standard portion, (2) the same as the standard portion, and (3) more than 1.5 times the standard portion. Food intake was categorized into nine frequencies (never, one to three times/month, one to two times/week, three to four times/week, five to six times/week, once/day, two to three times/day, four to six times/day, and seven times/day). The validation of this FFQ has been confirmed by comparing to the intake with 28-day weighted dietary records (DRs) as reference in a sub-cohort of the JPHC Study [24,25,26]. Using the Japanese Fifth Revised and Enlarged Edition of the Standard Tables of Food Composition (Fifth FCT), daily nutrient intakes were calculated [27].

However, an acrylamide database has been developed, which was established on measured values of acrylamide content in common Japanese foods [28,29,30,31,32,33,34,35]. This database was applied to assess acrylamide intake from the Japanese diet. The 5th FCT included 1878 food items. Of these items, there were 282 acrylamide-containing foods, 1276 nonacrylamide-containing foods, and 320 unclassified foods. Moreover, 39 heat-treated food items were classified as acrylamide-containing foods because acrylamide content varied according to the cooking method, even in the same food. As a result, acrylamide-containing foods added up to 321 food items (17% of the total number of food items), and they were used to assess acrylamide intake from DRs [28]. The acrylamide database was developed based on the linkage between the list of foods and the measured values of acrylamide content [29,30,31,32,33,34,35].

Of the 138 food and beverage items in the FFQ, 28 (20.3%) items were classified as acrylamide-containing foods. Most nutrient intakes in the FFQ were calculated depending on the intake of raw food; however, the level of acrylamide content varied with cooking methods. To calculate the acrylamide intake from the FFQ, the cooking methods and heated proportions of the following food items, as estimated from the DR, should be considered: rice (boiled, toasted, or stir-fried), vegetables (broccoli, bean sprouts, snap beans, sweet pepper, onion, squash, and cabbage), starchy vegetables (potato and sweet potato), and fried batter.

The validity and reproducibility of dietary acrylamide intake measurement from the FFQ of five-year follow-up survey have been reported elsewhere in the JPHC study [28]. In brief, the mean dietary acrylamide intake estimated from the FFQ for Cohorts I and II were 7.03 μg per day (standard deviation [SD] = 4.30) and 7.14 μg per day (SD = 3.38), while the mean intake estimated from DRs were 6.78 μg per day (SD = 3.89) and 7.25 μg per day (SD = 3.33), respectively. Deattenuated correlation coefficients were calculated between the FFQ and DRs for energy-adjusted dietary acrylamide intake. It ranged from 0.48 to 0.54 in Cohort I and from 0.37 to 0.40 in Cohort II, which showed a low-to-moderate correlation between the FFQ and DRs. Weighted kappa coefficients were over 0.80, which indicated a high degree of coincidence. Therefore, acrylamide intake measurement from the FFQ is suitable for use in the JPHC study [28].

### 2.3. Follow-Up and Identification of Cancer Cases

The residential status per year was confirmed by the residential registry. The follow-up period was defined from the beginning of the five-year follow-up survey until December 31, 2013. Of these participants, 5022 (5.9%) moved away from the targeted study area, and 13,756 (16.1%) died during the study period.

Data that from a link between major local hospitals and population-based cancer registries were used to confirm newly diagnosed ML, MM, and leukemia. The cases were coded according to the hospital-based cancer registries in Japan, using the International Classification of Diseases for Oncology, Third Edition. The morphology codes were 959 to 972 and 974 to 975 for ML, 973 and 976 for MM, 980 to 994 for leukemia. Death certificates were utilized as well as a supplementary source of information on outcome. We grouped ML cases by histologic subtypes, and further studied diffuse large-cell lymphoma (DLCL), follicular lymphoma (FL), and ML not otherwise specified [16]. Regarding histologic subtypes in leukemia, we focused on cases of acute lymphoblastic leukemia, acute myeloid leukemia (AML), and chronic myelogenous leukemia [36]. Adult T-cell leukemia/lymphoma (ATLL) is a relatively rare T-cell leukemia/lymphoma in Western countries, while is most common in parts of Japan, which has been linked to infection with human T-cell lymphotropic virus type 1. Thus, we discussed ATLL separately based on the clinical condition and incidence in Japan. In addition, myelodysplastic syndromes (MDS) have also been studied because they are considered to be a variable risk of transformation to acute leukemia [37]. Table 1 shows the detailed distribution of histologic subtypes of hematological malignancies in the JPHC Study with the morphology codes according to the World Health Organization classification [16].

### 2.4. Statistical Analysis

The cumulative number of person-years per subject was counted from the start of the five-year follow-up survey to the end of the follow-up, date of hematological malignancy diagnosis, date of departure from the study area, or date of death from any cause, depending on which occurred first. The median follow-up period was 16.0 years.

The adjustment of acrylamide intake by energy intake was made using the residual method. Subjects were divided into tertiles (T1, T2, and T3 groups) according to energy-adjusted acrylamide intake. To determine the relationship between ML, MM, leukemia, and tertiles of energy-adjusted acrylamide intake from the diet, we applied Cox proportional hazards models with T1 as the reference group for calculating hazard ratios (HRs) and 95% confidence intervals (CIs). With the assignment of ordinal values to tertiles of energy-adjusted acrylamide intake, trends were assessed. The characteristics of nondietary and dietary variables were compared among tertiles T1, T2, and T3 using the Kruskal–Wallis test or analysis of variance for continuous variables and using chi-square test for categorical variables as appropriate.

The adjusted variables in the multivariable model were gender, age (continuous), area (nine public health center areas and National Cerebral and Cardiovascular Center in Suita), body mass index (<19, 19 to <21, 21 to <23, 23 to <25, 25 to <27, 27 to <30, 30 to 40, or missing), smoking status (never, former, current, or missing), alcohol intake (nondrinker, <150 g/week, ≥150 g/week, or missing), physical activity (quartiles), occupation (professional or office worker, sales clerk or other, farmer, manual laborer, unemployed, or missing), and energy-adjusted consumption of fiber, carbohydrates, niacin, saturated fatty acid, mono unsaturated fat, and poly unsaturated fat (quintiles). Those variables were considered as potential confounding factors based on a previous study [16]. In the sensitivity analysis, subjects diagnosed with hematological malignancies within the first 3 years of follow-up were excluded.

Compared with nonsmokers, smokers have four times more acrylamide hemoglobin adducts. Its levels represent the exposure levels of acrylamide [38]. To clarify the effect of the interaction, we tested significance of the interaction term between acrylamide intake (tertiles) and smoking status and conducted subgroup analysis according to the following categories: never smokers, ever smokers (including former and current smokers), severally. Statistical analyses were performed using Stata/MP 16 (Stata Corp., College Station, TX, USA). All tests were two-tailed, and the significance level was set at *p* < 0.05 when comparing outcomes among the three groups, and *p* < 0.20 were considered statistically significant when assessing the interaction effect [39,40].

Furthermore, we analyzed DLCL, FL, ML not otherwise specified, AML, ATLL, and MDS in the multivariable-adjusted model to examine the dose–response relationship, with acrylamide as a continuous variable per 10 μg per day intake only because of the limited number of cases.

## 3. Results

After excluding participants who were not eligible, the total number of subjects included was 85,303 (including 39,982 men and 45,321 women) in this research. Until December 31, 2013, 326, 126, and 224 new cases of ML, MM, and leukemia had been identified, respectively.

Table 1 shows the distribution of histologic subtypes in hematological malignancies in the JPHC study. 

### 3.1. Analysis of Characteristics

Table 2 presents the characteristics of the 85,303 participants according to energy-adjusted acrylamide intake. The median (interquartile range, IQR) daily acrylamide intake (µg/day) were 3.58 (1.45) for T1, 6.09 (1.34) for T2, and 10.11 (3.67) for T3. In Japanese population, coffee (27.4%), green tea (21.6%), potatoes and starches (11.0%), vegetables (10.8%), and biscuits and cookies (10.6%) mainly contributed to the total acrylamide intake. Compared with the lowest acrylamide consumption group (T1), the highest acrylamide consumption group (T3) was characterized by a higher consumption of coffee, green tea, potatoes and starches, vegetables, biscuits and cookies, and meat but less fish. Moreover, the T3 group comprised more women, younger subjects, while the mean of body mass index, the proportions of heavy drinkers (≥150 g/week) and ever smokers were lower.

### 3.2. Association between Dietary Acrylamide Intake and Hematological Malignancy Risk

Table 3 shows the associations between daily acrylamide intake and ML. The results did not support that the daily acrylamide intake was associated with ML overall (HR for T2, 1.07; 95% CI: 0.82–1.40; HR for T3, 0.87; 95% CI 0.64–1.18). The HR for 10 µg per day increment of acrylamide intake was 0.98 (95% CI, 0.94–1.01). In the sensitivity analysis, the results were not different from the overall analysis. The HRs for acrylamide intake did not differ by smoking status (*p* for interaction = 0.38). For never smokers, the HR of the T3 group was lower than that of the T1 group; nevertheless, the difference was not significant (HR, 0.72; 95%CI, 0.48–1.10). The risk of ever smokers also did not increase with increasing acrylamide intake (*p* for trend = 0.48).

Table 4 displays the association of dietary acrylamide intake with the risk of MM. Generally, acrylamide intake was not related with an increased risk of MM. Although the HR of the T3 group was 0.64 (95% CI, 0.38–1.05) in multivariable-adjusted model, which was likely to lower than that of the T1 group, the result was not statistically significant. The interaction term between acrylamide intake and smoking status did not show significant difference (*p* for interaction = 0.24), and null associations were observed in the analyses for both never and ever smokers.

Table 5 presents the association between dietary acrylamide intake and leukemia. There were no significant associations observed in the overall (HR for T2, 1.01; 95% CI: 0.73–1.40; HR for T3, 1.01; 95% CI 0.71–1.45; *p* for trend = 0.94) or sensitivity analyses (HR for T2, 1.04; 95% CI: 0.73–1.49; HR for T3, 1.03; 95% CI 0.70–1.52; *p* for trend = 0.89). The HRs for acrylamide intake differed by smoking status, representing *p*-value for interaction was 0.13. For never smokers, the risks for T2 (HR, 0.82; 95%CI, 0.53–1.27) and T3 (HR, 0.86; 95%CI, 0.53–1.37) were lower than those for T1, although without significance. For ever-smokers, with increasing acrylamide intake, the risks of leukemia for T2 (HR, 1.42; 95% CI, 0.78–2.59) and T3 (HR, 1.33; 95% CI, 0.69–2.59) were slightly higher, although without any significant difference.

Regarding the histological subtypes, we did not observe any dose-response relationships between acrylamide intake, as a continuous variable per 10 μg per day, and the risk of DLCL, FL, ML NOS, AML, ATL, or MDS (data not shown). Null associations did not change in the analyses for both never and ever smokers.

## 4. Discussion

Our results imply that daily dietary acrylamide intake may not be related to ML, MM, or leukemia in middle-aged Japanese adults. To the best of our knowledge, the present study is the first population-based large-scale prospective cohort study in the world to examine the relationship between dietary acrylamide intake and leukemia as well as the first among Asian countries to examine the associations of dietary acrylamide intake with ML and MM. A null relationship was still observed in the stratification analysis by smoking status.

In the Alpha-Tocopherol, Beta-Carotene Cancer Prevention (ATBC) Study conducted in Finland among male smokers, a null association was found between dietary acrylamide intake and the risk of lymphomas [15]. However, histological subtypes of lymphatic malignancies were not further analyzed in that study. In contrast, in the Netherlands Cohort Study on Diet and Cancer (NLCS), researchers found that a higher dietary acrylamide intake may increase the risk of MM in all men (HR, 1.14; 95% CI: 1.01–1.27) and in never smoking men (HR, 1.98; 95% CI: 1.38–2.85) as well as the risk of FL in all men (HR, 1.28; 95% CI: 1.03–1.61) per 10 μg acrylamide per day as continuous increment [16]. In contrast, no associations were observed in our study, as reflected by HR = 0.98 (95% CI: 0.91–1.05) for MM in all men, HR = 0.97 (95% CI: 0.86–1.09) for MM in never smoking men, and HR = 1.03 (95% CI: 0.88–1.19) for FL in all men. Because of the relatively limited number of cases in both the previous NLCS study (MM: N = 170 in all men, N = 23 in never smoking men; FL: N = 42 in all men) and the present study (MM: N = 67 in all men, N = 29 in never smoking men; FL: N = 14 in all men), caution should be exercised while interpreting these conflicting results.

The null result for the dose-response effect of acrylamide intake from the diet on the risk of MM and FL was different from the NLCS result, and this may be partly explained by the low intake of acrylamide in the Japanese population. The mean daily intake of acrylamide in the JPHC study was 6.89 μg per day and 0.13 μg per day per kg weight, which was much lower than that reported in the NLCS (23 μg per day and 0.29 μg per day per kg weight) [16]. However, both studies reported the relative values of acrylamide by assessing the FFQ. In the future, the examination of acrylamide intake using hemoglobin adducts, which are considered biomarkers of internal dose, may provide a relatively accurate comparison.

There has been a series of previous studies in the JPHC to assess the relationship between acrylamide intake from the Japanese diet and the risk of cancers, targeting female cancers, digestive system cancers, lung cancer, liver cancer, and pancreatic cancer, and a null association was consistently indicated [9,10,11,12,13,14]. Our findings support those of previous studies, demonstrating that dietary acrylamide intake may not be related to the risk of hematological malignancies in the Japanese population.

The present research has some limitations to be discussed. First, the FFQ of the JPHC Study was composed by a limited list of foods and beverages. Thus, compared with the 24-h recall method, the estimation of dietary intake was less accurate. Second, the FFQ did not provide detailed information on temperature or time according to different cooking methods, which may influence upon the acrylamide content of foods. It could have also affected some nondifferential misclassification of the acrylamide intake, which would then likely have led to underestimation compared to the true risks. Third, occupational exposure is considered as another source of exposure to acrylamide [41]. However, the detailed information on occupational acrylamide exposure was not available in the JPHC study. Further studies are needed to elucidate the effect of dietary acrylamide exposure after considering the effect of occupational exposure.

Although there were some limitations with the use of FFQ to assess dietary acrylamide exposure, this is the achievable way for assessment in a large study population over a long period. Moreover, the validity and reproducibility of the JPHC FFQ for acrylamide intake have been studied [28]. Finally, the measurement of acrylamide consumption and information on covariates were provided only once. During the relatively study period, the participants might have changed their consumption levels of acrylamide. Thus, we should interpret the results with caution. However, the large size of the study population and long-term follow-up of the JPHC Study have some important strengths. First, the follow-up of the individuals was complete; thus, selection bias was unlikely in this study. Second, recall bias was also unlikely due to the prospective cohort design. Third, the large study size and long-term follow-up period enabled us to further examine specific histological subtypes of ML and leukemia. Subtypes are considered to differ in their etiology; therefore, they may differ in their relation to dietary acrylamide intake.

## 5. Conclusions

Our research is the first epidemiological study to determine the relationship between dietary acrylamide intake and the risk of lymphatic malignancies among Asians as well as the first to investigate the association with leukemia. The findings of this research demonstrate that dietary acrylamide intake at a relatively low level may not be associated with the risk of hematological malignancies in the Japanese population. Further studies using biomarkers, which could provide the internal dose of acrylamide, are required to assess the carcinogenic action of acrylamide exposure in humans.

## Figures and Tables

**Table 1:** Distribution of histologic subtypes in hematological malignancies in the Japan Public Health Center-based (JPHC) Study.

Histologic Subtype	ICD-O-3	N
Malignant lymphomas, all ^a,b^	959–972, 974–975	326
Diffuse large-cell lymphoma ^c^	9675, 9680, 9684	123
Follicular lymphoma ^c^	9690–9698	29
T-cell lymphoma or Mucosis fungoides	9700–9709, 9714	18
Extranodal marginal B-cell lymphoma or MALT	9699	18
Mantle cell lymphoma	9673	4
Walden-ström’s macroglobulinemia and immunocytoma	9671, 9761	3
Burkitt’s lymphoma	9687	2
Lymphoblastic lymphoma	9727–9728	1
Malignant lymphoma NOS ^c^	9590–9596	80
Hodgkin lymphoma	9650–9669	10
Multiple myeloma (MM) ^b^	973, 976	126
Leukemia, all ^a,b^	980–994	224
Acute lymphoblastic leukemia	9832–9835	11
Acute myeloid leukemia ^c^	9840, 9861, 9866–9867, 9873–9875, 9891, 9896	63
Chronic myeloid leukemia	9863, 9875	18
Other specific subtypes ^d^		
Adult T-cell leukemia/lymphoma ^c^	9827	77
Myelodysplastic syndromes (MDS) ^c^	9980, 9982–9983, 9985, 9987–9989	53
Chronic lymphocytic leukemia	9670, 9823	7

Abbreviations: ICD-O-3, International Classification of Diseases for Oncology, third edition; MALT, mucosa-associated lymphoid tissue; NOS, not otherwise specified. ^a^ Only case numbers for subtypes with a sufficient number of cases are listed. Thus, subgroups do not add up to all. ^b^ N = cases available for analyses by tertiles, and the results are shown in tables. ^c^ N = cases available for analyses by continuously modeled dietary acrylamide intake (per 10 μg/d), and the results are mentioned only in the manuscript. ^d^ Other specific subtypes are listed separately based on the clinical circumstances.

**Table 2:** Characteristics of participants (*n* = 85,303) in the JPHC study.

Characteristics	Tertile of Energy-Adjusted Acrylamide Intake						
	Tertile 1		Tertile 2		Tertile 3		*p*-Value ^d^
Number of Participants	28,435		28,434		28,434		
Men, %	57.6		44.3		38.8		
Dietary variables							
Acrylamide intake							
Range, μg/d	0.00–4.84		4.85–7.67		7.68–66.68		
Median, ^a^ μg/d	3.58	(1.47)	6.09	(1.37)	10.11	(3.66)	
Median, ^a^ μg/kg body weight·d	0.06	(0.03)	0.11	(0.03)	0.18	(0.08)	<0.001
Total energy intake, ^a^ kcal/d	1902.6	(864.4)	1916.1	(824.3)	1904.3	(824.8)	0.015
Coffee, ^a^ g/d	17.8	(67.6)	83.3	(139.6)	210.1	(325.1)	<0.001
Green tea, ^a^ g/d	223.0	(372.2)	405.9	(513.2)	575.6	(848.2)	<0.001
Vegetables, ^a^ g/d	156.5	(129.5)	197.0	(144.9)	202.0	(153.6)	<0.001
Fruit, ^a^ g/d	140.3	(170.1)	185.7	(185.8)	184.5	(186.8)	<0.001
Meat, ^a^ g/d	48.4	(48.8)	50.5	(43.7)	50.2	(42.4)	<0.001
Fish, ^a^ g/d	75.3	(62.2)	80.3	(58.0)	75.1	(55.8)	<0.001
Biscuits, ^a^ g/d	0.3	(1.4)	1.4	(2.3)	2.0	(5.8)	<0.001
Potato, ^a^ g/d	7.5	(11.0)	13.8	(18.4)	15.1	(21.4)	<0.001
Fiber, ^a^ g/d	10.6	(5.7)	12.6	(6.0)	12.9	(6.3)	<0.001
Carbohydrates, ^a^ g/d	254.3	(58.7)	261.0	(49.4)	265.0	(49.0)	<0.001
Niacin, ^a^ mg/d	16.6	(5.9)	18.1	(5.3)	19.2	(5.3)	<0.001
Saturated fatty acid, ^a^ g/d	14.4	(8.1)	15.5	(6.9)	16.4	(6.9)	<0.001
Mono unsaturated fat, ^a^ g/d	16.8	(8.0)	18.3	(7.0)	19.1	(6.9)	<0.001
Poly unsaturated fat, ^a^ g/d	11.5	(4.7)	12.5	(4.3)	12.6	(4.3)	<0.001
Nondietary variables							
Age at Five-year follow-up study, ^a^ y	58.0	(11.0)	57.0	(13.0)	55.0	(13.0)	<0.001
Body mass index, ^b,c^ kg/m^2^	23.7	(3.1)	23.6	(3.0)	23.4	(3.0)	<0.001
Smoking status, %							
Never smoker	58.0		64.9		64.0		<0.001
Former smoker	10.6		8.3		6.8		
Current smoker	25.0		21.0		23.5		
Missing	6.4		5.8		5.6		
Number of cigarettes/d, ^a,c^ only for current smokers	20.0	(9.0)	20.0	(10.0)	20.0	(15.0)	<0.001
Alcohol intake, %							
Nondrinker	45.6		54.9		59.8		<0.001
<150 g/week	17.8		20.7		22.7		
≥150 g/week	34.5		20.4		15.3		
Missing	2.1		2.1		2.2		
Physical activity (METs), %							
Quartile 1	22.0		22.4		23.4		<0.001
Quartile 2	18.1		19.9		20.2		
Quartile 3	19.1		22.4		23.5		
Quartile 4	18.3		18.3		18.1		
Missing	22.6		16.9		14.8		
Diabetes, % yes	8.3		6.7		5.4		<0.001
Occupation, %							
Professional or office worker	15.9		16.2		17.0		<0.001
Sales clerk or other	26.6		27.1		30.1		
Farmer	18.7		15.0		12.6		
Manual laborer	10.6		14.9		15.5		
Unemployed	9.4		8.2		6.9		
Missing	18.7		18.7		18.0		

^a^ Median, interquartile range. ^b^ Mean, standard deviation. ^c^ Number of individuals with missing data of the following parameters: body mass index *n* = 2163; number of cigarettes per day for current smokers *n* = 378. ^d^ Kruskal–Wallis test was applied to skew distributed continuous variables, analysis of variance was applied to normally distributed continuous variables, and chi-square test was applied to categorical variables.

**Table 3:** Hazard ratios (95% confidence intervals) for malignant lymphoma according to tertile of acrylamide intake.

	Quartile of Energy-Adjusted Acrylamide Intake									
	10 μg/d		Tertile 1 (Lowest)		Tertile 2		Tertile 3 (Highest)			*p* for Interaction ^d^
	HR	(95% CI)	HR	(95% CI)	HR	(95% CI)	HR	(95% CI)	*p* for Trend	
Number of subjects	85,303		28,435		28,434		28,434			
Person-years	1,267,766		417,242		424,878		425,646			
Number of malignant lymphoma	326		117		119		90			
Crude rate (per 100,000)	25.7		28.0		28.0		21.1			
Gender-, age-and area-adjusted model ^a^	0.98	(0.95–1.01)	1.00	(Reference)	1.07	(0.83–1.39)	0.89	(0.67–1.19)	0.47	
Multivariable model 1 ^b^	0.98	(0.94–1.01)	1.00	(Reference)	1.07	(0.82–1.40)	0.87	(0.64–1.18)	0.40	0.38
Multivariable model 1 (excluding cases <3 years)	0.98	(0.94–1.01)	1.00	(Reference)	1.09	(0.82–1.45)	0.86	(0.62–1.19)	0.39	
By smoking status										
Never smoker										
Number of subjects	53,136		18,137		18,102		16,897			
Person-years	817,851		252,824		284,192		280,835			
Number of malignant lymphoma	176		67		65		44			
Crude rate (per 100,000)	21.5		26.5		22.9		15.7			
Multivariable model 1	0.99	(0.95–1.04)	1.00	(Reference)	0.93	(0.65–1.33)	0.72	(0.48–1.10)	0.14	
Ever smoker ^c^										
Number of subjects	27,082		8475		8663		9944			
Person-years	382,535		140,840		118,384		123,311			
Number of malignant lymphoma	129		41		47		41			
Crude rate (per 100,000)	33.7		29.1		39.7		33.2			
Multivariable model 1	0.96	(0.92–1.01)	1.00	(Reference)	1.40	(0.91–2.18)	1.19	(0.73–1.93)	0.48	

Abbreviations: 95% CI = 95% confidence intervals. ^a^ Gender-, age (continuous)- and area-adjusted model. ^b^ Multivariable Model 1 additionally adjusted for: body mass index (<19, 19 to <21, 21 to <23, 23 to <25, 25 to <27, 27 to <30, 30 to 40, or missing), smoking status (never, former, current, or missing), alcohol intake (nondrinker, <150 g/week, ≥150 g/week, or missing), physical activity (quartiles), occupation (professional or office worker, sales clerk or other, farmer, manual laborer, unemployed, or missing), and energy-adjusted consumption of fiber, carbohydrates, niacin, saturated fatty acid, mono unsaturated fat, and poly unsaturated fat (quintiles). ^c^ Ever smoker was defined as former and current smokers. ^d^ *p* value for the interaction term between acrylamide intake (tertiles) and smoking status (never, former, current, or missing).

**Table 4:** Hazard ratios (95% confidence intervals) for multiple myeloma according to tertile of acrylamide intake.

	Quartile of Energy-Adjusted Acrylamide Intake									
	10 μg/d		Tertile 1 (Lowest)		Tertile 2		Tertile 3 (Highest)			*p* for Interaction ^d^
	HR	(95% CI)	HR	(95% CI)	HR	(95% CI)	HR	(95% CI)	*p* for Trend	
Number of subjects	85,303		28,435		28,434		28,434			
Person-years	1,267,766		417,242		424,878		425,646			
Number of multiple myeloma	126		49		48		29			
Crude rate (per 100,000)	9.9		11.7		11.3		6.8			
Gender-, age- and area-adjusted model ^a^	0.98	(0.93–1.03)	1.00	(Reference)	0.99	(0.66–1.48)	0.65	(0.41–1.04)	0.09	
Multivariable model 1 ^b^	0.98	(0.93–1.03)	1.00	(Reference)	0.99	(0.65–1.50)	0.64	(0.38–1.05)	0.09	0.24
Multivariable model 1 (excluding cases <3 years)	0.98	(0.93–1.04)	1.00	(Reference)	1.00	(0.63–1.58)	0.66	(0.38–1.14)	0.15	
By smoking status										
Never smoker										
Number of subjects	53,136		18,137		18,102		16,897			
Person-years	817,851		252,824		284,192		280,835			
Number of multiple myeloma	76		28		29		19			
Crude rate (per 100,000)	9.3		11.1		10.2		6.8			
Multivariable model 1	0.99	(0.93–1.05)	1.00	(Reference)	1.00	(0.58–1.73)	0.70	(0.37–1.34)	0.29	
Ever smoker ^c^										
Number of subjects	27,082		8475		8663		9944			
Person-years	382,535		140,840		118,384		123,311			
Number of multiple myeloma	38		17		12		9			
Crude rate (per 100,000)	9.9		12.1		10.1		7.3			
Multivariable model 1	0.97	(0.88–1.07)	1.00	(Reference)	0.72	(0.33–1.57)	0.52	(0.21–1.27)	0.15	

Abbreviations: 95% CI = 95% confidence intervals. ^a^ Gender-, age (continuous)- and area-adjusted model. ^b^ Multivariable Model 1 additionally adjusted for: body mass index (<19, 19 to <21, 21 to <23, 23 to <25, 25 to <27, 27 to <30, 30 to 40, or missing), smoking status (never, former, current, or missing), alcohol intake (nondrinker, <150 g/week, ≥150 g/week, or missing), physical activity (quartiles), occupation (professional or office worker, sales clerk or other, farmer, manual laborer, unemployed, or missing), and energy-adjusted consumption of fiber, carbohydrates, niacin, saturated fatty acid, mono unsaturated fat, and poly unsaturated fat (quintiles). ^c^ Ever smoker was defined as former and current smokers. ^d^ *p* value for the interaction term between acrylamide intake (tertiles) and smoking status (never, former, current, or missing).

**Table 5:** Hazard ratios (95% confidence intervals) for leukemia according to tertile of acrylamide intake.

	Quartile of Energy-Adjusted Acrylamide Intake									
	10 μg/d		Tertile 1 (Lowest)		Tertile 2		Tertile 3 (Highest)			*p* for Interaction ^d^
	HR	(95% CI)	HR	(95% CI)	HR	(95% CI)	HR	(95% CI)	*p* for Trend	
Number of subjects	85,303		28,435		28,434		28,434			
Person-years	1,267,766		417,242		424,878		425,646			
Number of leukemia	224		82		74		68			
Crude rate (per 100,000)	17.7		19.7		17.4		16.0			
Gender-, age- and area-adjusted model ^a^	1.00	(0.97–1.04)	1.00	(Reference)	0.92	(0.67–1.26)	0.87	(0.62–1.21)	0.39	
Multivariable model 1 ^b^	1.02	(0.98–1.05)	1.00	(Reference)	1.01	(0.73–1.40)	1.01	(0.71–1.45)	0.94	0.13
Multivariable model 1 (excluding cases <3 years)	1.02	(0.99–1.06)	1.00	(Reference)	1.04	(0.73–1.49)	1.03	(0.70–1.52)	0.87	
	
By smoking status										
Never smoker										
Number of subjects	53,136		18,137		18,102		16,897			
Person-years	817,851		252,824		284,192		280,835			
Number of leukemia	128		48		40		40			
Crude rate (per 100,000)	15.7		19.0		14.1		14.2			
Multivariable model 1	1.01	(0.97–1.06)	1.00	(Reference)	0.82	(0.53–1.27)	0.86	(0.53–1.37)	0.51	
Ever smoker ^c^										
Number of subjects	27,082		8475		8663		9944			
Person-years	382,535		140,840		118,384		123,311			
Number of leukemia	69		23		24		22			
Crude rate (per 100,000)	18.0		16.3		20.3		17.8			
Multivariable model 1	1.03	(0.97–1.09)	1.00	(Reference)	1.42	(0.78–2.59)	1.33	(0.69–2.59)	0.38	

Abbreviations: 95% CI = 95% confidence intervals. ^a^ Gender-, age (continuous)- and area-adjusted model. ^b^ Multivariable Model 1 additionally adjusted for: body mass index (<19, 19 to <21, 21 to <23, 23 to <25, 25 to <27, 27 to <30, 30 to 40, or missing), smoking status (never, former, current, or missing), alcohol intake (nondrinker, <150 g/week, ≥150 g/week, or missing), physical activity (quartiles), occupation (professional or office worker, sales clerk or other, farmer, manual laborer, unemployed, or missing), and energy-adjusted consumption of fiber, carbohydrates, niacin, saturated fatty acid, mono unsaturated fat, and poly unsaturated fat (quintiles). ^c^ Ever smoker was defined as former and current smokers. ^d^ *p* value for the interaction term between acrylamide intake (tertiles) and smoking status (never, former, current, or missing).

## Data Availability

Not Applicable.
